# Genetics of Microenvironmental Sensitivity of Body Weight in Rainbow Trout (*Oncorhynchus mykiss*) Selected for Improved Growth

**DOI:** 10.1371/journal.pone.0038766

**Published:** 2012-06-11

**Authors:** Matti Janhunen, Antti Kause, Harri Vehviläinen, Otso Järvisalo

**Affiliations:** 1 MTT Agrifood Research Finland, Biometrical Genetics, Jokioinen, Finland; 2 Finnish Game and Fisheries Research Institute, Laukaa, Finland; University of Queensland, Australia

## Abstract

Microenvironmental sensitivity of a genotype refers to the ability to buffer against non-specific environmental factors, and it can be quantified by the amount of residual variation in a trait expressed by the genotype’s offspring within a (macro)environment. Due to the high degree of polymorphism in behavioral, growth and life-history traits, both farmed and wild salmonids are highly susceptible to microenvironmental variation, yet the heritable basis of this characteristic remains unknown. We estimated the genetic (co)variance of body weight and its residual variation in 2-year-old rainbow trout (*Oncorhynchus mykiss*) using a multigenerational data of 45,900 individuals from the Finnish national breeding programme. We also tested whether or not microenvironmental sensitivity has been changed as a correlated genetic response when genetic improvement for growth has been practiced over five generations. The animal model analysis revealed the presence of genetic heterogeneity both in body weight and its residual variation. Heritability of residual variation was remarkably lower (0.02) than that for body weight (0.35). However, genetic coefficient of variation was notable in both body weight (14%) and its residual variation (37%), suggesting a substantial potential for selection responses in both traits. Furthermore, a significant negative genetic correlation (−0.16) was found between body weight and its residual variation, i.e., rapidly growing genotypes are also more tolerant to perturbations in microenvironment. The genetic trends showed that fish growth was successfully increased by selective breeding (an average of 6% per generation), whereas no genetic change occurred in residual variation during the same period. The results imply that genetic improvement for body weight does not cause a concomitant increase in microenvironmental sensitivity. For commercial production, however, there may be high potential to simultaneously improve weight gain and increase its uniformity if both criteria are included in a selection index.

## Introduction

Early phases of selective breeding can generate rapid genetic responses in farmed animals. This typically involves genetic improvement of mean performance in the direction of selection. It is well established that many concurrent improvements in animal husbandry, including nutrition, housing and veterinary practices, accompany the genetic enhancement in animal performance. Additionally, trait heterogeneity can evolve over time, for example via increased or reduced susceptibility of individuals to variable and unmeasured microenvironmental factors. Understanding the genetic basis of such concurrent changes in quantitative traits reveals how selection influences the ability of individuals to respond to unpredictably fluctuating environmental conditions via developmental mechanisms, and helps us to explain the persistence of phenotypic variability within populations.

Microenvironmental sensitivity refers to an individual’s ability to be buffered against local non-specific environmental factors (e.g., fluctuating weather, light conditions and food supply, and competitive social interactions) and subtle developmental noise, and it is considered synonymous to developmental instability [1]–[3]. Microenvironmental sensitivity of a genotype can be quantified by the amount of residual variation in a trait expressed by the genotype’s offspring within a (macro-)environment the offspring share. In modern quantitative genetic analysis, residual variance can be best estimated using an animal model which partitions a phenotype of an individual into its additive genetic and residual components, the latter being the part left unexplained by genetics and systematic fixed effects such as gender, age and management treatments [4], [5]. In farm animal husbandry, increased residual and thus phenotypic variation is disadvantageous because it hampers the efficiency of production throughout the supply chain from producers to consumers [6], [7]. Moreover, large size variation in rearing groups promotes the formation of behavioral dominance hierarchies which reduce animal welfare and elevate mortality [8]–[10]. This can be partly avoided by active size sorting and grouping of animals. Currently, there is increasing interest to investigate to what extent residual variation can be genetically reduced by animal breeding programmes.

Permanent changes in microenvironmental sensitivity are possible only when there is additive genetic variation for residual variation. In other words, different genotypes should produce differently variable progenies. The recent evidence from both wild and farmed animals imply that genotypes indeed differ in their amount of residual variation of traits [11]. Even though heritability of residual variation is generally low, it can be exploited to increase uniformity by direct selection [12]–[16]. Further, it has been suggested that intense directional selection for a trait (mean) value can lead to increased residual (and thus phenotypic) variation because the extreme individuals with a higher selection probability are also the genotypes passing down high variability [14], [17], [18]. This would be worrisome because selection would make individuals more sensitive to their environment. The counter hypothesis is that during adaptation to an environment, either in the wild or in human-controlled conditions of farmed species, microenvironmental sensitivity is decreased due to the adaption to a focal environment [19], [20].

Previous work has concentrated on terrestrial vertebrates and laboratory model species, which greatly differ from aquatic species, and from salmonids in particular. Salmonids have a multitude of characteristics that make the genetic analysis of microenvironmental sensitivity in growth important. In aquaculture production, new populations and species are constantly introduced in intensive captive breeding, providing an opportunity to investigate the genetic effects of artificial selection (or domestication process [21]) on both the trait mean value and its underlying variation. Furthermore, salmonids exhibit an extraordinary polymorphism and diversity in morphological, behavioral and life-history traits, including alternative growth, migration and reproduction strategies expressed across and within single populations [22]–[25]. Some of these responses are adaptive responses to the highly stochastic natural conditions. Salmonids also display strong dominance hierarchies, especially within farmed populations, in which few individuals can defend food resources, increasing phenotypic variation in growth [26]–[28]. Given that fish as ectotherms are particularly sensitive to varying ambient conditions that can influence ontogenetic trajectories, individual differences in growth are more pronounced in fish compared to farmed terrestrial animals. For example, in cultured salmonids, phenotypic coefficient of variation (CV) of body weight varies between 20–40% [29], whereas in chicken and pigs it is around 10–15% [30]–[32]. Finally, an additional strength of using salmonids to study genetic architecture of microenvironmental sensitivity is that the established breeding programmes generate large number of families in successive generations, and due to their high fecundity, high family sizes can be produced, both factors needed for an effective genetic analysis of residual variation.

To investigate the inheritance of microenvironmental sensitivity and its genetic responses across generations when directional selection is performed for improved growth, we analyzed multigenerational pedigreed data covering ten year classes and 46 546 individuals from the Finnish breeding programme for rainbow trout, *Oncorhynchus mykiss* (Walbaum). We first estimated the proportion of genetic variation in residual variation for body weight in fish being maintained in the same location. By providing a common macroenvironment across year classes and by using the animal model, we ensured that residual variation can be regarded as microenvironmental sensitivity (or developmental stability) that results from non-systematic environmental factors and internal developmental noise. Second, we estimated the genetic correlation between the additive genetic effects for body weight and its residual variation. Finally, by estimating genetic trends that quantify genetic responses across multiple generations, we investigated the effects of selective breeding for body weight on the genetic change in microenvironmental sensitivity.

## Methods

### Ethics Statement

All procedures involving animals were approved by the animal care committee of the Finnish Game and Fisheries Research Institute (FGFRI).

### Data Source

The data originated from the Finnish national rainbow trout breeding programme maintained by the FGFRI and MTT Agrifood Research Finland. The breeding nucleus is held at the Tervo Fisheries Research and Aquaculture station in Central Finland (63°1′ N, 26°39′E).

The phenotypic data included 45 900 records of body weight from individuals born during 1992–2002 and reared at the same freshwater nucleus station. The fish represented eight year classes and belonged to two subpopulations with four successive generations (Pop I and Pop IIa) [33], [34]. Each year class consisted of 94–270 full-sib families established from matings of 37–90 sires with 92–270 dams. The subpopulations share a common genetic base from which the founding individuals were sampled in 1989 for PopI and in 1990 for PopII. Even though the base population was preceded by a long-term cultivation background, only the studied generations belong to a systematic breeding programme in which intensive genetic selection based on estimated breeding values has been practiced. The pedigree information extended over the five generations and comprised 46 546 individuals, including the 364 base population animals without phenotypic observations.

The generation interval of the study population was 3–4 years. Annual selection of breeding candidates was made using a multitrait selection index with main emphasis on improved growth The selection index has consisted of best linear unbiased predictions of breeding values for body weight measured at the age of 2 and 3 years (since 1992), maturity age (since 2001) [35], and body shape, skin color and its spottiness (since 2001) [36]. Parental fish were mated in spring using either nested paternal hierarchical or partial factorial designs [33].

Full-sib egg batches were incubated separately, and at the eyed-egg stage, they were transferred to one or two 150-liter indoor family tanks. Hatching of eggs occurred in June. During the following winter, after six months of growing in the family tanks, equal amount of fingerlings (of 50–100 g body weight) from each family tank were haphazardly sampled and individually tagged with passive integrated transponders (Trovan, Ltd., Ulm, Germany) and then transferred to a flow-through earth-bottomed raceway at the Tervo station. The fish were fed with commercial dry feed. In Finland, year is highly seasonal and the effective growing season lasts from early May to late October.

After the second growing season, the two-year-old fish were individually weighed to the nearest 1 g (mean 1020±315 (SD) g, *n* =45 900). The number of individuals within each year class ranged between 2 518–10 753. The proportion of sexually matured (2+) males in the entire data-set was 14.9%, whereas no mature females were found.

To improve the reliability of genetic parameters for residual variation, only sire families with at least 35 offspring (*n* =457 sires) were selected for the analysis. Large family sizes are needed to obtain accurate and unbiased genetic parameters and estimated breeding values (EBVs) for residual variation [14].

### Genetic Analysis

The estimation of genetic parameters and genetic trends was conducted using a bivariate animal model [31]. The ASReml 3.0 software applying restricted maximum likelihood (REML) was used [37]. The first trait was body weight for which a linear mixed ‘mean model’ was fitted:
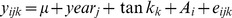
(1)where 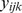 is body weight of an individual *i*, *µ* is the overall population mean, 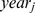 is the fixed effect of birth year (*j* =8 years), 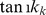 is the random interaction effect between birth year and common environment shared by full-sibs before tagging (*k* = family tan *k* × year number), 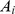 is the random genetic animal effect with a pedigree (*i* = number of animals), and 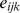 is the residual error term with separate variance 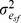 for each sire family *sf*. The common environment effect is modeled without the pedigree information. The values for Akaike’s Information Criteria (AIC) [38] and Bayesian Information Criteria (BIC) [39] were lower for the model with heterogeneous residual variance structure, suggesting a better fit to the data compared to the model with homogeneous residual variance (AIC: 539554 and 542160; BIC: 541235 and 542171, respectively).

The second trait was microenvironmental sensitivity which was quantified by the log-transformed squared residual values, 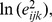 obtained from the mean model (1) and used as new observations in the ‘variance model’. Log-transformed squared residual values quantify the contribution of each individual to population’s residual variation [15], [31], [32], [40]. In contrast to sire-dam models, the residuals of an animal model include only unexplained environmental and developmental noise, and they are not confounded by the additive genetic Mendelian sampling term. The animal ‘variance model’ was:
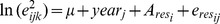
(2)where 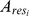 is the genetic effect of animal *i* for 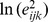 and 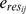 is the random residual effect. For the random effects of 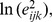 the assumptions were 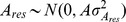 and 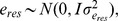 where **A** is the additive genetic relationship matrix with additive genetic variance 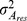 and **Ι** is the identity matrix with homogeneous residual variance 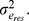 The random effect for common environment × birth year was omitted from the variance model because its variance explained less than 2% of the total phenotypic variance and it did not significantly differ from zero.

Because the residuals of the model 1 are used as an input variable for the model 2, the model for the mean and the residual variation was iteratively solved by conducting 30 consecutive bivariate analyses. At each iterative round, 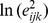 for the variance model were updated with residuals from the previous round’s mean model. The residuals 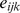 and 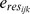 were assumed to follow a bivariate normal distribution and be uncorrelated (i.e., their residual covariance was set to zero). The convergence criteria within separate runs were fulfilled when the REML log-likelihood changed less than 0.002 × iteration number and the individual variance parameter estimates changed less than 1% between successive iterations [37].

### Calculation of Genetic Parameters and Genetic Trends

Heritability of weight mean was calculated as 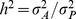 and the common environment effect ratio as 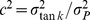 using the variance components from model 1. Here 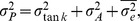 where 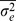 is the average residual variance of sire families. In addition to common environment effects of full sibs, 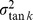 may include parts of non-additive genetic and maternal variance. Genetic coefficient of variation was calculated as 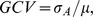 where *µ* is the phenotypic mean of the population. *GCV* describes the propensity of the trait to respond to selection, that is, its evolvability [41].

Heritability of residual variation was calculated as 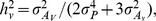 where 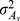 is the transformed additive genetic variance of residual variation from model 2 and 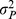 is the phenotypic variance of body weight obtained from model 1 [14]. The genetic variance 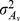 was calculated as 
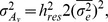
 where 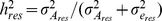 is the heritability of 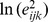 and 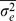 is the average residual variance obtained from model 1. Genetic coefficient of variation for residual variation was calculated as: 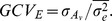 An estimate of genetic correlation between the additive genetic effects for body weight and its residual variance was obtained from the bivariate analysis where direct estimation of co-variance between the two traits is possible.

The approximate standard errors for estimated variance components and variance ratios were calculated using ASReml. The standard error of 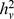 was approximated according to Mulder *et al.* [31].

REML log-likelihood values and the parameter estimates for body weight were found to remain relatively stable across the 30 iterative rounds, whereas 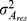 oscillated. Therefore, the results from bivariate analysis are presented as averages of all ASReml runs (*n*=30 rounds). The observed oscillation is inherent to the statistical model used and is mainly due to an interplay between 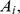 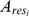 and the residual 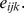 An increase in 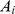 causes a decrease in the residual and thereby lowers 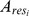 (and *vice versa*).

To investigate whether or not genetic changes in mean body weight and its mircroenvironmental sensitivity occurred during selective breeding, genetic trends were determined for both traits and for both subpopulations separately. The genetic trends were obtained by plotting the average estimated breeding values (i.e., the predicted genetic levels for 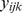 and 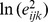 obtained from individuals’ averages across the 30 iterative rounds) against the birth year of fish.

## Results

### Genetic Variation

Heritability for body weight was moderate (0.35), whereas the common environment ratio was low (0.05) (Table 1). Genetic coefficient of variation for body weight was slight (0.14). Heritability estimate of residual variation was low (0.02), though it was greater than its standard error (Table 2). Yet, the moderately high genetic coefficient of variance for residual variation (*GCV_E_*=0.37) suggests that there is notable genetic potential in microenvironmental sensitivity of body weight.

**Table 1:** Estimates of variance components and variance ratios (± approximate standard errors) for body weight.

Parameter^a^	Estimate
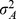	20 888 (1515)
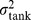	3 089 (286)
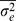	35 674 (7444)
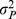	59 652 (7439)
*h* ^2^	0.350 (0.051)
*c* ^2^	0.052 (0.009)
*GCV*	0.142

a additive genetic variance; 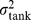 common environment variance; 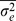 the average residual variance of sire families; 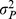 phenotypic variance; 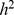
– heritability, 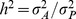; 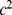
– common environment effect ratio, 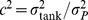; 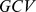
– coefficient of genetic variation, 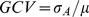.

**Table 2:** Estimated variance components and variance ratios (± approximate standard errors) for microenvironmental sensitivity of body weight.

Parameter^a^	Estimate
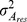	0.374 (0.028)
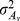	1.81 E + 08
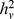	0.024 (0.006)
*GCV_E_*	0.376

a additive genetic variance in ln(e^2^) (model 2);

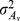
 – transformed genetic variance in the quantitative genetic model for genetic heterogeneity of residual variation [13], 
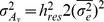
; 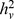 – heritability, 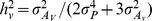;

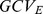
– genetic coefficient of variation, 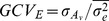.

### Genetic Correlation between Body Weight and its Residual Variation

There was a slight but significant negative genetic correlation between body weight and its residual variation (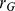
=−0.157±0.039 (S.E.)), indicating that high body weight was genetically associated with decreased microenvironmental sensitivity.

### Genetic Trends

**Figure 1:**
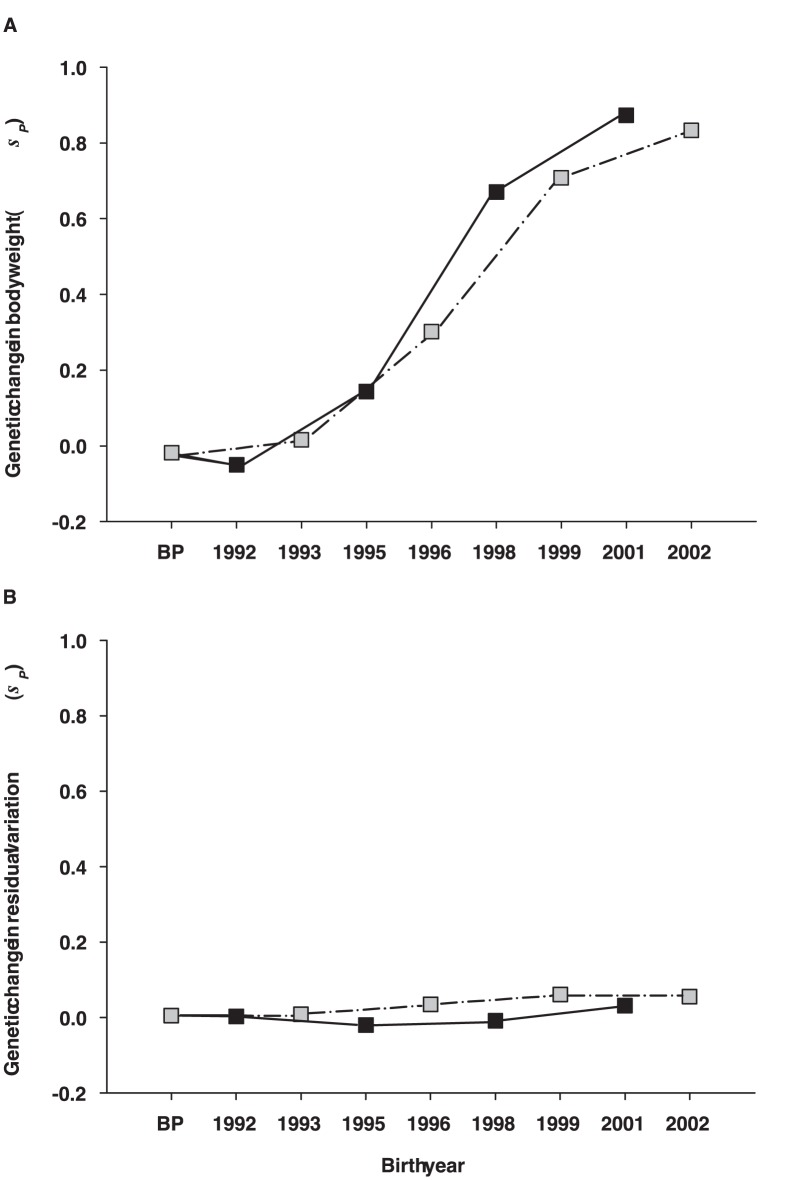
Genetic changes in mean and residual variation of body weight. Average genetic changes for A) body weight mean and B) its microenvironmental sensitivity in two subpopulations (black and grey box) of rainbow trout. The averages are given in the units of phenotypic standard deviation (*σ*_P_).

Body weight showed a clear genetic improvement during the study period. Over the four generations of selection, the cumulative genetic gains in the two sub-populations were 199 g to 208 g, corresponding to an average of 0.83 increase in phenotypic standard deviation or 5.5% per year (Fig. 1a). In contrast, mean estimated breeding values for microenvironmental sensitivity remained stable across the year classes (Fig. 1b).

## Discussion

### Low Heritability but Moderate Evolvability for Microenvironmental Sensitivity

We found a low 0.02 heritability estimate for residual variation of body weight (i.e., microenvironmental sensitivity) in 2-year-old rainbow trout. This is in marked contrast with the moderate heritability of 0.35 for body weight. The low heritability estimate of microenvironmental sensitivity is somewhat surprising as large within- and between-family variation in fish growth is created by multiple factors, some of them presumably exhibiting substantial genetic variation. However, the notable genetic coefficient of variation (37%, when genetic variation for residual variance is scaled by average residual variance) indicates the presence of substantial additive genetic variation for microenvironmental sensitivity. Regarding most life-history traits, the low heritabilities yet paradoxically high evolvability are attributed to the high residual variation accumulating from the variable underlying physiological and behavioral traits [41], [42]. Similarly, body weight and its variation can be influenced by many underlying component traits such as feeding behavior, feed utilization and metabolism [43].

There are several factors that can maintain genetic variation in microenvironmental sensitivity in the population under study. First, high initial growth rates and energy resources are related to increased probability of early maturation in salmonids [44], [45]. Likewise, in rainbow trout, rapid growth is genetically and phenotypically correlated to early maturity age [35], [46], [47]. In our population, male fish primarily mature at ages of 2 to 3 years. Maturity age in males has a moderate heritability of 0.23–0.34, and thus there are family differences in the frequency of maturing individuals [33], [35]. This alone may create genetic variation in microenvironmental sensitivity: high residual variation would be found in families with both early and late maturing individuals, and low residual variation in families with either only early or only late maturing individuals. Accordingly, it was logical not to include maturation as a fixed factor in the statistical model because sexual development itself captures part of the within-family variation we were interested in. Second, following the former reasoning, the genetic variation observed for resistance and/or tolerance to parasite-mediated cataract (*Diplostomum* spp.) in our population may create genetic variation in microenvironmental sensitivity. Some families remain uninfected while others have both infected and uninfected individuals, and the infected individuals exhibit reduced growth [48]. Third, social interactions associated with behavior and growth differences have also been found to create additional genetic variation in chicken and pigs [49], [50], and presumably in fish as well [51]. A large proportion of the genetic variation underlying socially affected traits remains hidden, i.e., is not accounted for by the direct heritability estimates, and can thus only be revealed by unexplained residual variation. Last, it is important to recall that even though the genetic characteristics of farmed fish populations are influenced by life histories originating from their wild ancestors, the results from a genetic analysis of farmed populations cannot be extrapolated back to wild populations [52]. Nevertheless, the estimates of genetic parameters obtained from farmed populations help us to understand biologically meaningful phenomena and also advance general knowledge of the factors underlying phenotypic variation in quantitative traits [53].

Although rainbow trout, among other salmonids, possess a capacity of considerable growth and life-history strategy variation both across and within families [33], [35], [54], the observed heritability estimate for residual variation in body weight is of similar low magnitude that has been reported for less variable terrestrial animals [11], [14]. Correspondingly, *GCV*_E_ was in the range of those found in chickens, mice, pigs and rabbits (25–50%) [11]. Fluctuating asymmetry, the degree of random non-directional deviations between morphological characteristics measured from left and right hand side of individuals, is an alternative measure of developmental instability. In accordance with the original idea by Lerner [55], increased heterozygosity has been found to reduce fluctuating asymmetry in bilateral traits of both wild and farmed rainbow trout [56], [57]. However, the estimated low heritability for fluctuating asymmetry led the authors to conclude that dominance effects have a major contribution to the control of developmental stability [58]. Developmental instability is often assumed to be selectively disadvantageous due to the increased risk of drift from the phenotypic optimum [3], [59], [60], but empirical support for this view is largely inconclusive [61]. It is probable that in some cases, such as the morphological traits of plants, selection favors increased sensitivity as a bet-hedging strategy [62].

It is possible that similar to life-history traits [63]–[66], developmental stability is inherently an important fitness correlate, and the strong directional selection during the long history of animals has led to its low heritability [58]. Meanwhile, many underlying environmental and genetic factors affecting microenvironmental sensitivity retain its genetic coefficient of variation at a moderate level. Nevertheless, further analyses are needed to test whether the genetic parameters show similar values in wild fish populations or when fish populations are in their first generations of domestication. The methods developed by animal breeders and also used here [31], [32] can be applied to wild populations when pedigree information is established using molecular genetic markers.

### Direct and Correlated Responses to Selection in Microenvironmental Sensitivity

The low heritability estimate observed here does not necessarily indicate that microenvironmental sensitivity would be weakly responsive to selection. Heritability, the ratio of additive genetic variance to phenotypic variance, is one predictor of genetic potential to selection responses, though in this context, genetic coefficient of variation provides a more reasonable measure of evolvability, similar to *GCV* for trait means [41], [67]. In our study, *GCV*_E_ was over two times higher than *GCV* for body weight, suggesting a good opportunity to obtain reduction in random environmental variation by selection.

Some selection experiments and breeding programmes have obtained considerable genetic responses in traits with low heritability (e.g., developmental stability in *Drosophila* [68], [69]; piglet survival [70]), supporting the idea that also the amount of residual variation can be modified by selection. Similarly, residual variation is expected to be reduced by 10% after one generation of selection when it is included in a selection index along with the phenotypic trait value [71]. To effectively breed for a trait with a low heritability, phenotypic records from a large number of relatives are required. Controlled matings and large family sizes inherent to rainbow trout and many other aquaculture species enhance the estimation of breeding values with moderate accuracy [14], [72].

To our knowledge, this study is the first multigenerational breeding experiment on aquatic organisms to assess the correlated genetic effect of strong directional selection on microenvironmental sensitivity of a trait. The genetic correlation between body weight and its residual variation was negative, implying that a high trait value was linked to a slightly reduced microenvironmental sensitivity. This combined with the low heritability of residual variation predicts only a weak decreasing microenvironmental sensitivity across successive generations in response to selection for rapid growth. However, the genetic trend for microenvironmental sensitivity remained stable or slightly elevated over the course of the selection period, while body weight mean displayed a 6% genetic increase per generation. These results together indicate that genetic improvement of body weight does not make rainbow trout more sensitive to microenvironmental perturbations. This is important animal welfare issue, because increase in size heterogeneity would lead to serious challenges in animal husbandry.

Intense mass selection based on individuals’ own phenotype is expected to increase phenotypic variation within a population even when there is no additive genetic correlation between trait and its residual variation [14], [18]. Moreover, studies on salmonid fish suggest that selection for rapid growth may indirectly select for competitive ability and aggressiveness, thus increasing the likelihood for increased size variation in farmed fish during the breeding process [73]–[76]. The observed patterns in genetic trends do not conform to these assumptions. Referring to the former proposition, however, a multitrait selection method in our study population was not only based on the phenotypic information of an individual itself but also the performance of its all relatives was taken into account. This makes the predictions concerning responses in environmental variation more difficult. Nevertheless, the negative genetic correlation between the body weight and its microenvironmental sensitivity could be expected to counterbalance, to some extent, the rate of increase in growth variation due to scale effects. Previous studies on terrestrial animals have shown that the genetic correlation between quantitative traits and their residual variations can vary from negative to positive, depending on the species and trait analyzed [31], [40], [77]–[79]. Similar inconsistent results have been found in selection experiments. For example, Ibáñez-Escriche *et al.* [16] demonstrated a decrease in phenotypic CV of body weight traits in mice selected for increased growth. In contrast, long-term selection experiments on *Drosophila* fruitfly showed that phenotypic variation can be substantially higher in the lines selected for high and low abdominal bristle number relative to the unselected base population [80], [81].

In conclusion, heterogeneity of residual variation in rainbow trout growth was found to be partly under genetic control. This implies the possibility for selection to favor genotypes with low variability when constancy across microenvironmental conditions is important. The negative genetic relationship between body weight and its microenvironmental sensitivity presumably facilitates improving weight gain and simultaneously increasing its uniformity/robustness if both objectives are incorporated into a selection index. In addition, increasing the growth potential of fish does not seem to cause a concomitant change in the trait’s microenvironmental sensitivity.
